# Perception survey of crisis and emergency risk communication in an acute hospital in the management of COVID-19 pandemic in Singapore

**DOI:** 10.1186/s12889-020-10047-2

**Published:** 2020-12-17

**Authors:** Lai Meng Ow Yong, Xiaohui Xin, Jennifer Mei Ling Wee, Ruban Poopalalingam, Kenneth Yung Chiang Kwek, Julian Thumboo

**Affiliations:** 1 Medical Social Services, Singapore General Hospital, Singapore, 169608 Singapore; 2 Health Services Research Unit, Singapore General Hospital, Singapore, 169608 Singapore; 3 Communications, Singapore General Hospital, Singapore, 169608 Singapore; 4 Medical Board, Singapore General Hospital, Singapore, 169608 Singapore; 5 Chief Executive Office, Singapore General Hospital, Singapore, 169608 Singapore

**Keywords:** Emergency risk communication, Risk communication, COVID-19, Public health crises

## Abstract

**Background:**

Emergency risk communication is a critical component in emergency planning and response. It has been recognised as significant for planning for and responding to public health emergencies. While there is a growing body of guidelines and frameworks on emergency risk communication, it remains a relatively new field. There has also been limited attention on how emergency risk communication is being performed in public health organisations, such as acute hospitals, and what the associated challenges are. This article seeks to examine the perception of crisis and emergency risk communication in an acute hospital in response to COVID-19 pandemic in Singapore and to identify its associated enablers and barriers.

**Methods:**

A 13-item Crisis and Emergency Risk Communication (CERC) Survey, based on the US Centers for Disease and Control (CDC) CERC framework, was developed and administered to hospital staff during February 24–28, 2020. The survey also included an open-ended question to solicit feedback on areas of CERC in need of improvement. Chi-square test was used for analysis of survey data. Thematic analysis was performed on qualitative feedback.

**Results:**

Of the 1154 participants who responded to the survey, most (94.1%) reported that regular hospital updates on COVID-19 were understandable and actionable. Many (92.5%) stated that accurate, concise and timely information helped to keep them safe. A majority (92.3%) of them were clear about the hospital’s response to the COVID-19 situation, and 79.4% of the respondents reported that the hospital had been able to understand their challenges and address their concerns. Sociodemographic characteristics, such as occupation, age, marital status, work experience, gender, and staff’s primary work location influenced the responses to hospital CERC. Local leaders within the hospital would need support to better communicate and translate hospital updates in response to COVID-19 to actionable plans for their staff. Better communication in executing resource utilization plans, expressing more empathy and care for their staff, and enhancing communication channels, such as through the use of secure text messaging rather than emails would be important.

**Conclusion:**

CERC is relevant and important in the hospital setting to managing COVID-19 and should be considered concurrently with hospital emergency response domains.

**Supplementary Information:**

The online version contains supplementary material available at 10.1186/s12889-020-10047-2.

## Background

The WHO [1] defines emergency risk communication as an intervention to enable everyone at risk to take informed decisions to protect themselves, their families and communities against threats to their survival, health and well-being. It is a critical component in emergency planning and response [2, 3] and its role and importance has been acknowledged increasingly around the world. Its inclusion as one of the eight core public health capacities in the International Health Regulations (2005), which WHO member states are required to develop, strengthen and maintain, underscores the global acknowledgement of its growing role and importance [4–6]. The release of the first ever evidence-based guidance on policy and practice of emergency risk communication from the WHO also highlights the importance of emergency risk communication practice [7].

This expanding interest in emergency risk communication is not surprising given how emerging infectious diseases have taken centre-stage globally in recent years. Global disease outbreaks such as the Ebola virus outbreak in West Africa in 2014–2015, Zika virus syndrome over various regions of the world in 2015–2016, and yellow fever outbreak in Africa in 2016, highlighted major gaps and challenges in emergency risk communication during epidemics and other public health emergencies [8]. The recent coronavirus (COVID-19) pandemic, which was first identified in Wuhan China, December 2019, also accentuates the need for effective communication in emergency response [9–11].

Despite its importance and growing institutional interest in it, studies on emergency risk communication systems, mechanisms and detailed practices remain limited [12–14]. Existing studies on enablers and barriers of emergency risk communication have focused primarily at public or national level. Evidence shows that knowledge and attitudes towards public health threat are influenced by socioeconomic position and interpersonal networks, and could differ across various segments of population, which can impact compliance with recommended behaviours in emergency risk communication [14]. Studies reveal timely release of information and trust in leaders and government are positively associated with the adoption of recommended behaviours during a public health crisis [11, 14, 15]. Managing and sharing information can help strengthen partnerships, and social networking sites could empower people and communities in terms of informed decision-making [16, 17].

Conversely, an absence of these enablers poses barriers to emergency risk communication in public health crisis. It could result in confusion and delays with regard to policy decisions aimed at stemming disease transmission [18]. For example, a lack of trust in the messenger would mean that people are less likely to adopt recommended behaviours; previous mistrust in leaders and government could influence communication effectiveness [11, 14]. A lack of accurate and evidence-based information, top-down communication or oversaturation of competing messages may also lead to increased fears and an attitude of information avoidance and non-compliance to protocols [11, 19]. Rumours, misperceptions and misinformation, whether on social networking sites or otherwise could hinder disease prevention efforts [12, 17]. Poor communication may lead to avoidable cases of the viral disease and may damage country’s economy and reputation [12].

While these findings could lend insight to how they may apply in acute hospitals, arguably microcosms of the larger public or community, there needs to be an expanded and a specific body of knowledge to inform emergency risk communication policy, practice and strategies at hospital level [20, 21]. There has generally been limited attention on how emergency risk communication is being performed in public health organisations, such as acute hospitals, and what the associated challenges are [20, 21]. Emergency risk communication is crucial in hospital emergency response, as it can have direct impact on its healthcare staff, patient care and wider healthcare delivery at the national level. More importantly, outbreaks of any emerging diseases predominately affect healthcare workers, as they come into closest contact with infected persons [15]. This also underscores the importance of looking at emergency risk communication in hospitals.

This research study thus aims to examine the perception of crisis and emergency risk communication (CERC) in an acute hospital in response to COVID-19 in Singapore and to identify the associated enablers and barriers. It looks specifically at the Singapore General Hospital (SGH), the largest acute hospital in Singapore, with 1700 beds. Founded in 1821, SGH is the public sector’s flagship hospital. It is part of the Singapore Health Services, a healthcare cluster/system of hospitals, national specialty centres and primary care clinics. Every year, SGH sees over 1 million patient visits, and together with the other national specialty centres located on the SGH Campus, offer a comprehensive range of clinical specialties. As an academic healthcare institution, SGH is a key teaching hospital for doctors, nurses and allied health professionals in the national medical education programmes and conducts translational and clinical research. Building on its experience with the Severe Acute Respiratory Syndrome (SARS) epidemic from March 12 to June 4 in 2003 [22], SGH is currently one of the primary hospitals caring for COVID-19 patients in Singapore.

At the point when this research was conducted, Singapore reported 89 confirmed COVID-19 cases, where 38 of them were in hospital, five were in critical condition. The first confirmed COVID-19 case in Singapore presented at SGH on 23 January 2020. The numbers then quickly climbed, with new clusters of COVID-19 cases being identified in the community within a short span of time. By the end of this research study, the situation remained fluid, with SGH reporting 8 confirmed cases in the hospital. As little was known about the COVID-19 virus, the hospital had to work quickly in terms of its crisis and emergency risk communication, to ensure the safety of its patients, healthcare staff and the community. At the time of writing of this article, there were a total of 57,915 cases in Singapore and crisis communication remained important to handling this pandemic.

This research study seeks to contribute to the body of knowledge on hospital CERC and inform how emergency risk communication can be better enhanced, particularly as the COVID-19 pandemic continues to evolve. It will help inform subsequent phases (maintenance and resolution) of the CERC framework in terms of communication strategies in the management of COVID-19. Findings from the study may also have wider relevance to hospitals and countries elsewhere in the CERC of public health crises.

## Methods

### Study design

In designing an appropriate survey to examine enablers and barriers of emergency risk communication in an acute hospital, we used the US CDC CERC framework. The CERC is an evidence-based framework that helps anyone who communicates on behalf of an organisation in response to a public health emergency. It is widely adopted for strategic communication in risk and crisis situations [16]. The CERC “encompasses the urgency of crisis communication, empower decision-making, and is communicated by an expert that may be a participant” [23]. This integrated framework of risk and crisis communication is based on CERC six main principles: be first, be right, be credible, express empathy, promote action and show respect [24]. It consists of four phases (preparation, initial, maintenance, and resolution), where each has its own associated tasks [23].

As the development of COVID-19 was at a relatively initial phase at the time of the study, this survey focused on the first two CERC phases, which are ‘preparation’ and ‘initial’. The first phase ‘preparation’ emphasizes the importance of drafting and testing messages, developing partnerships, creating plans, and determining approval process. The second phase ‘initial’ involves expressing empathy, explaining risks, promoting action, and describing response efforts [3].

A 13-item CERC survey with questions revolving around the two phases and associated tasks of the CERC was developed for this study after several rounds of iteration with inputs from experts in emergency risk communications, health services research, sociology and social work to establish content validity. We tested the acceptability of the questions with diverse hospital staff of various ranks from Singapore General Hospital (SGH), where this research study was conducted. It involved interviews with a sample of 45 staff, where we tested the face validity of the survey, and conducted cognitive interviews to refine the layout and wording of the survey. Following the pilot, the final version of the survey was prepared, which comprised 13 close-ended questions, and an open-ended question to solicit qualitative feedback on the risk communication strategies (see Additional file 1: CERC Survey). The survey instrument has good internal consistency, with a Cronbach alpha coefficient of 0.97. There was no total scoring to assess the overall perceived hospital COVID-19 CERC for the two phases; the CERC is also not designed for this purpose. The survey questions were framed based on the hospital context.

### Participants

Given that emergency response in a hospital involves all staff, including those who were directly and indirectly involved in patient care, the electronic version of the CERC survey was put up on the hospital intranet homepage on 24 February 2020 and it closed on 28 February 2020. All staff were encouraged to participate. The response to each of the 13 questions in the form of statements about different aspects of the communication strategies was measured using a ‘1–5’ Likert scale, where ‘1’ is ‘strongly disagree’ to ‘5’ which is ‘strongly agree’. Some of the statements included were: “The regular updates from SGH on the COVID-2019 situation are understandable and actionable”, “The crisis communication plans are clear so far”, and “I am sufficiently engaged in the preparedness planning”.

### Analysis

All survey data collected were aggregated for statistical analysis. The analysis, using IBM SPSS Statistics software version 25, examined the overall patterns and made comparisons across meaningful groups, such as occupation, gender, age, years of work experience in SGH, and marital status. We compared responses in terms of ‘strongly agree’ or ‘agree’ versus others except for ‘work experience in SGH’. The Pearson Chi-square test was used to compare the variables, and Yates’ Correction for Continuity was used to compensate for the overestimates of the chi-square value used in 2 × 2 table. We used an alpha level of 0.05 for all statistical tests. The qualitative feedback data were analysed thematically according to the CERC framework. Thematic analysis is an approach used for identifying, analyzing, organizing, describing, and interpretation of meaning or themes within qualitative data [25, 26]. We performed research triangulation and data collection triangulation to ensure the credibility of the study. The first and second authors analysed and coded the data separately, and then came together to discuss and finalise the themes. The results were then used to contextualise and corroborate the survey findings.

## Results

A total of 1154 people responded to the survey out of hospital staff strength of 9888 (or 11.7%). The largest proportion of respondents based on demographic characteristics were female (84.3%), Singaporean or Singapore Permanent Resident (88.2%), married (53.6%), and nurses (54.3%). The respondents were mostly staff with no supervisory roles (72.3%), had no direct contact with patients (73.9%), whose primary work location was in inpatient (non-isolation) wards (47.8%), and who were not directly involved in in treating COVID-19 patients (87.2%). The mean age of participants was 37.7 years (SD = 11.5).

A large proportion of them obtained their sources of information on hospital management of COVID-19 from SGH senior management updates, guidelines and instructions (77.6%), department emails (62.6%), and their supervisors (62.2%). Only 34.6% of them drew information and updates from social media (hospital Facebook Workplace Group) (Table 1).

**Table 1:** Respondent characteristics

Characteristics	Participants (***n*** = 1154)^a^
Age in years (Mean(SD))	37.71 (11.46)
Years in SGH (Mean(SD))	12.37 (10.25)
Gender (N(%))	
Female	973 (84.3)
Male	180 (15.6)
Nationality (N(%))	
Singaporean or Permanent Resident	1018 (88.2)
Malaysian	22 (1.9)
Filipino	63 (5.5)
Indian	25 (2.2)
Chinese	13 (1.1)
Myanmar	12 (1.0)
Others (e.g., Bangladeshi, Vietnamese, Irish)	1.0 (1.1)
Marital status (N(%))	
Single	475 (41.2)
Married	619 (53.6)
Separated or Divorced	27 (2.3)
Widowed	10 (0.9)
Occupation(N(%))	
Doctor	22 (1.9)
Nurse	627 (54.3)
Allied Health Professional	206 (17.9)
Administrative staff member	118 (10.2)
Ancillary support staff member - SGH employees	13 (1.1)
Ancillary support staff member –External staff contracted to work in SGH	167 (14.5)
Supervisory role (N(%))	
No	834 (72.3)
Yes	319 (27.6)
Sources of information and updates on the management of COVID-19 in SGH (N(%))	
SingHealth Staff Advisory	466 (40.4)
SGH Senior Management Updates, guidelines and instructions	895 (77.6)
Social media (e.g., SGH Facebook Workplace Group)	
Supervisor	399 (34.6)
SGH Middle Management (e.g., HOD, Divisional Directors)	718 (62.2)
Department emails	396 (34.3)
Colleagues and peers (e.g., chat groups, exchanges)	722(62.6)
Others (e.g., gov.sg WhatsApp, MOH website, CNA, news media, friends)	487 (42.2)35 (3.0)
Direct contact with patients (N(%))	
No	300 (26.0)
Yes	853 (73.9)
Primary work location since start of COVID-19 outbreak (N(%))	
Emergency Department	33 (2.9)
Inpatient - Isolation wards	33 (2.9)
Inpatient - Other clinical area(s)	552 (47.8)
Outpatient	266 (23.1)
Non-clinical area(s) (no patient contact)	268 (23.2)
Direct involvement in treating patients with confirmed or suspected COVID-19 (N(%))	
No	1006 (87.2)
Yes	147 (12.7)

^a^Because of missing data, denominators do not all equal 1154

### Perception of communication strategies

Overall, most of the respondents (close to 80%) responded to the survey questions with an ‘agree’ or ‘strongly agree’. Table 2 summarises the respondents’ responses to the survey questions. Most respondents found the hospital regular updates on the COVID-19 situation to be understandable and actionable (94.1%), and information released by hospital senior management to be accurate, concise and timely, and repeated enough to keep staff safe (92.5%). A large proportion of the respondents reported that the hospital had been clear in explaining the necessary actions they needed to take to keep themselves safe (92.7%). Only 79.4% of the respondents highlighted that SGH had been able to understand their challenges and to address their concerns during the outbreak. Of the respondents, 82.5% of them reported to be sufficiently engaged in preparedness planning, and 84.0% of them stated that platforms such as social media and emails provided useful avenues for sharing of information and feedback.

**Table 2:** Participants’ perception of the communication strategies used in hospital response to COVID-19 ((*N* = 1154)^a^

CERC Survey Statements	Respondents who ‘agree’ or ‘strongly agree’ to the statements N(%)
Q1. The regular updates from SGH on the COVID-19 situation are understandable and actionable	1086 (94.1)
Q2. SGH adequately prepares me for the challenges I am likely to face.	1047 (90.8)
Q3. The crisis communication plans are clear so far.	1032 (89.5)
Q4. SGH Senior Management possesses the necessary knowledge and expertise on the situation and has been consistent in the delivery of their message.	1049 (90.9)
Q5. The information released by SGH senior management has been accurate, concise, and timely, and are repeated enough to keep staff safe.	1067 (92.5)
Q6. My direct supervisor has consistently provided me with accurate, concise, and timely information for me to navigate in this disease outbreak.	975 (84.5)
Q7. I am sufficiently engaged in the preparedness planning.	952 (82.5)
Q8. SGH has been able to understand my challenges and address my concerns during this outbreak.	917 (79.4)
Q9. SGH has been able to provide explanations of the risks associated with the COVID-19 situation in a simple, concise, and direct manner.	1044 (90.5)
Q10. SGH has been clear in explaining the necessary actions I need to take to stay safe.	1069 (92.7)
Q11. I am clear about what SGH is doing in response to the COVID-19 situation.	1065 (92.3)
Q12. The constant updates from SGH Senior Management increase my trust in the credibility of the organisation.	1044 (90.5)
Q13. Platforms, such as emails and social media (Facebook @ Workplace group-Pneumonia(China)-Chat with CEO&CMB) provide useful avenues for sharing of information and feedback.	970 (84.0)

^a^Because of missing data, denominators do not all equal 1154

### Sociodemographic characteristics on CERC perceptions

Chi-square test for independence was used to compare the CERC responses with sociodemographic characteristics (Table 3). Findings revealed that supervisory role, patient contact, and involvement in treating COVID-19 patients did not differ by sociodemographic characteristics on CERC. There were no differences between CERC questions 1 and 2 and the sociodemographic characteristics. Significant relationships were found between some of the responses to the CERC survey and sociodemographic characteristics, such as occupation, age, work experience in SGH, primary work location, marital status, and gender. These are discussed as follows.

**Table 3:** Chi-square results for sociodemographic variables and SGH COVID-19 CERC survey questions (phase 1 and 2) questions

Characteristics (*p*, %)	Q3	Q4	Q5	Q6	Q7	Q8	Q9	Q10	Q11	Q12	Q13
Occupation	0.12	0.24	0.002	0.504	0.131	0.031	0	0.001	0	0.001	0.002
Doctors	77.3%	81.8%	72.7%	77.3%	68.2%	59.1%	59.1%	72.7%	63.6%	68.2%	59.1%
Others	89.8%	91.3%	92.9%	84.8%	82.8%	80.1%	91.2%	93.4%	93.0%	91.1%	85.1%
Primary work area	0.24	0.153	0.935	0.028	0.078	0.374	0.181	0.165	1	0.096	0.417
Inpatient	88.5%	89.9%	92.4%	86.9%	84.5%	80.7%	89.5%	91.9%	92.4%	89.3%	85.4%
Outpatient	90.8%	92.5%	92.7%	82.0%	80.3%	78.4%	91.9%	94.2%	92.5%	92.3%	83.5%
Direct involvement in treating COVID-19 or suspected COVID-19 cases	0.842	0.741	0.728	0.698	0.282	0.284	0.190	0.198	0.526	0.919	0.512
No	89.7%	91.2%	92.6%	84.5%	82.1%	79.2%	91.1%	93.3%	92.6%	90.7%	84.3%
Yes	89.1%	90.4%	91.8%	85.7%	85.7%	83.0%	87.7%	90.4%	91.2%	90.5%	86.4%
Age	0.012	0.132	0.016	0.317	0.082	0.037	0.226	0.143	0.189	0.004	0.032
> = 40	92.7%	92.9%	95.1%	86.1%	85.4%	83.1%	92.2%	94.6%	93.9%	94.1%	87.7%
Below 40	87.8%	90.1%	91.1%	83.7%	81.2%	77.8%	89.8%	92.1%	91.6%	88.7%	82.7%
Work experience in SGH (in years)	0.04	0.036	0.015	0.186	0.002	0.088	0.053	0.026	0.213	0.03	0.003
< 1	94.1%	92.2%	94.1%	88.0%	74.5%	84.3%	88.2%	90.2%	90.2%	90.2%	78.4%
1 < 5	90.9%	92.1%	94.5%	84.8%	84.8%	79.9%	94.2%	95.1%	92.7%	88.4%	81.1%
5 < 10	83.9%	86.7%	88.1%	81.1%	78.0%	76.2%	87.4%	89.2%	89.5%	87.1%	80.0%
> = 10)	91.6%	92.8%	94.0%	88.7%	87.1%	83.4%	91.8%	94.3%	93.6%	93.2%	88.6%
Marital status	0.54	0.316	0.139	0.536	0.224	0.001	0.007	0.227	0.141	0.266	0.358
Married	90.1%	91.9%	93.5%	85.5%	84.2%	83.7%	92.7%	93.8%	93.5%	91.6%	85.7%
Others	88.8%	90.0%	91.0%	84.0%	81.3%	75.7%	87.9%	91.8%	91.0%	89.5%	83.5%
Nationality	1	0.904	0.881	0.019	0.464	0.369	0.791	0.284	1	0.74	0.272
Singaporean or Permanent Resident	89.6%	91.1%	92.6%	83.7%	82.2%	79.2%	90.8%	93.3%	92.4%	90.6%	84.1%
Others	89.6%	91.8%	91.9%	91.9%	85.2%	83.0%	89.6%	90.4%	92.6%	91.9%	88.1%
Gender	0.969	0.467	0.74	0.795	0.273	0.111	0.465	0.563	0.23	0.107	0.001
Female	89.5%	91.5%	92.7%	84.5%	83.1%	80.5%	90.9%	93.2%	92.9%	91.3%	86.2%
Male	89.9%	89.4%	91.7%	85.6%	79.4%	75.0%	88.9%	91.7%	90.0%	87.2%	76.0%

*p ≤ *.05*

*+p* ≤ *.01*

#### Occupation

Nurses, allied health professionals and administrative staff, as a group, endorsed every aspect of hospital CERC questions at a significantly higher percentage than doctors. The magnitude of difference ranges from 14% (94.3% vs. 80.1%) to 27% (86.4% vs. 59.1%). They were more likely to endorse the following statements: (1) the information released by the hospital senior management had been accurate, concise and timely and were repeated enough to keep staff safe (92.9 vs. 72.7%; *p* = 0.002); (2) the hospital had been able to provide explanations of the risks associated to COVID-19 in a simple, concise and direct manner (91.2% vs. 59.1%; *p* < 0.001); (3) they were clear about what the hospital was doing in response to COVID-19 (93.0% vs. 63.6%; *p* < 0.001); (4) the hospital had been clear in explaining the necessary actions they needed to take to stay safe (93.4% vs. 72.7%, *p* = 0.001); (5) constant updates from hospital senior management increased their trust in the credibility of the organization (91.1% vs. 68.2%; *p* = 0.001); (6) platforms, such as emails and social media provided useful avenues for sharing of information and feedback (85.1% vs. 59.1%; *p* = 0.002); and (7) the hospital had been able to understand their challenges and address their concerns during this outbreak (80.1% vs. 59.1%; *p* = 0.03).

#### Age

There were significant relationships between perceived hospital CERC and respondents’ age. Specifically, older staff (age 40 and above) were more likely to report that hospital crisis communication plans were clear (92.7% vs. 87.8%; *p* = 0.01). Significantly higher percentage of them found the information released by the hospital senior management to be accurate, concise and timely and were repeated enough to keep staff safe (95.1% vs. 91.1%; *p* = 0.02). Older staff were also more likely to report that constant updates from the hospital senior management increased their trust in the credibility of the organization (94.1% vs. 88.7%; *p* < 0.01). A significant proportion of them found the hospital had been able to understand their challenges and address their concerns during the outbreak (83.1% vs. 77.8%; *p* = 0.04).

#### Marital status

Married staff were more likely to report that the hospital had been able to understand their challenges and address their concerns during the outbreak (83.7% vs. 75.7%; *p* = 0.001). A significantly higher percentage of them reported that the hospital had been able to provide explanations of the risks associated with the COVID-19 situation in a simple, concise and direct manner (92.7% vs. 87.9%; *p* = 0.007) as compared to non-married staff.

#### Work experience in SGH

There were significant relationships between perceived hospital CERC and respondents’ years of work experience in the organisation. Specifically, a lower percentage of them with ‘5 and more but less than 10 years of experience’ were more likely to report that crisis communication plans were clear (83.9%; *p* = 0.04). Those with less than 1 year of work experience were less likely to report that platforms, such as emails and social media were useful avenues for sharing of information and feedback (78.4%; *p* = 0.03).

Interestingly, a substantially lower percentage of staff with ‘5 and more but less than 10 years of experience’ endorsed several aspects of the hospital CERC statements. They were less likely to agree that (1) information released by the hospital senior management had been accurate, concise and timely and repeated enough to keep staff safe (88.1%; *p* = 0.015); (2) the hospital senior management possessed the necessary knowledge and expertise on the situation and had been consistent in the delivery of their message (86.7%, *p* = 0.036); (3) they were sufficiently engaged in the preparedness training (78.0%; *p* = 0.002); (4) the hospital had been clear in explaining the necessary actions they needed to take to stay safe (89.2%; *p* = 0.026); and that (5) constant updates from the hospital senior management increased their trust in the credibility of the organization (87.1%; *p* = 0.03).

#### Primary work location of staff

Staff who worked in inpatient areas were more likely to agree that their direct supervisor had consistently provided them with accurate, concise and timely information for them to navigate in this disease outbreak (86.9% vs. 82.0%; *p =* 0.028).

#### Gender

Females were more likely to report that platforms such as emails and social media provided useful avenues for sharing of information and feedback (86.2% vs. 76.0; *p* = 0.001).

### Qualitative themes

Five themes emerged from the open-ended question to explore how the hospital could better support them and address their concerns. There was a total of 251 responses. Overall, the findings were generally positive. However, there were communication challenges at local leadership or middle management levels and in executing resource reallocation plans, and on human resource and staff welfare matters and general communication approaches.

#### Accurate, concise and timely information as enabler (*n* = 72)

Most respondents reported favourably that the hospital had been clear in its instructions and the daily routine instructions were helpful. Many felt that the hospital was reasonably well-prepared and that it had done enough in its communication. For example, a respondent (P132) stated: “Current measures and communication channel are good enough to support and address concerns if any”. Another (P198) highlighted: “SGH is doing a good job. With constant updates and contact with nurses and other healthcare workers to the ground, this boosts the energy to keep us giving our best contribution to our patients”. These results support the quantitative findings, where 92.5% of the respondents indicated that information released by the hospital senior management had been accurate, concise and timely, and were repeated enough to keep them safe.

#### Local leadership and middle management as barrier (*n* = 18)

Some respondents reported challenges at local leadership or middle management level. They stated that a lack of clear communication from direct supervisors to staff to translate some of the hospital updates to actionable plans created anxiety in them. They were also less adequately engaged and there was low level of empathy from the local leaders and middle management (i.e., direct supervisors, head of departments). Respondent 11 asserted: “Direct supervisors need to be more in touch and show concern for staff”.

#### Resource reallocation and logistics as barriers (*n* = 33)

Some respondents raised the need for clearer instructions to ensure staff safety, particularly for those involved in patient screening at thermal scanner points. “The schedule for screening is not communicated properly and neither were the details of changes in the screening hours and the change of screening location were discussed”, highlighted respondent 56. They reported that the schedule for screening of patients and visitors at various entrances and exits in the hospital had to be communicated properly. Respondents suggested that as COVID-19 would likely be here for some time, it would be necessary to communicate plans that describe a reasonable mechanism that would not tax existing volunteers, who were hospital staff with other duties. Others reported that meeting mechanisms in the hospital could be improved, and there could be clearer communication on viable, secure and acceptable alternatives on how staff could carry out meetings instead of having face-to-face meetings. “To me, meeting mechanisms should be improved. We need clear instructions in terms of how to organize our meetings to ensure safety of meeting attendees”, stated respondent 61.

#### Human resource and staff welfare as barrier (*n* = 72)

More than a quarter of the respondents indicated that communication of instructions in terms of human resource policies and staff welfare related to COVID-19 could be improved. For example, respondents perceived communication of ‘no leave taking’ instruction to be unreasonable, lacking empathy and argued that clearer explanations would be necessary. “In the area of manpower management and leave freezes, it could have been made clearer right at the start. Moving forward, it would be good to document and work out a better plan regarding leave and leave freezes when the next emergency happens”, asserted respondent 63. Staff welfare, including for those who were not directly involved in the care of COVID-19 patients should also be looked into, as the overflow effects in terms of roles and duties would mean they were indirectly supporting the hospital in this endeavour. “All healthcare workers play a part in day-to-day operations in hospital regardless of whether patient fronting or not patient fronting. There should be a fair appreciation for all staff”, stated respondent 129. Additionally, they indicated that there would be a need to psychologically support staff to better adhere to social segregation and distancing precautionary measures and this needs to be communicated.

#### Communication as barrier (*n* = 72)

More than a quarter of the respondents who provided qualitative feedback felt that the hospital could provide clearer instructions, instead of just rules, and to have a go-to-person for any clarification on procedures and protocols related to COVID-19. They reported that an anonymous feedback channel would be useful to improve communication. Some suggested clearer explanations on hospital priorities in terms of balancing the disease outbreak measures and ensuring continuity of service would be necessary. For example, respondent 82 stated: “Senior management needs to communicate better to staff about the hospital’s general strategy for working, what the priorities are, how they envision balancing disease outbreak measures against continuing to provide care and other essential services to patients”. Other respondents reported being overwhelmed by the barrage of information and stated that urgent messages should be sent via secure text messaging application(s), rather than email, which could provide more real-time updates, instructions and directions on the COVID-19 development in the hospital.

## Discussion

This research study has described the perception of crisis and emergency risk communication in an acute hospital in response to COVID-2019 in Singapore. Analysis of the findings revealed that most respondents reported regular updates, which is a key component in CERC [27] by the hospital were understandable, actionable, accurate, concise and timely. Updates from the hospital senior management were the preferred source of information on COVID-19 for the respondents. There was also a strong sense of trust in the credibility of the hospital among staff, and staff were clear about how to keep themselves safe in combatting COVID-19. Significant relationships however were reported between the perception of the hospital CERC, and occupation, age, work experience in the hospital, primary work location of staff, marital status, and gender. Qualitative findings revealed that the CERC cuts across hospital emergency domains, involving human resource, staff welfare, local leadership and resource reallocation. These are discussed below.

In this study, nurses, allied health professionals and administrative staff were more likely to report favourably in many areas of the hospital CERC. However, some groups, such as doctors, staff below 40 years of age, non-married, located in the outpatient setting, and with ‘5 to less than 10 years of work experience’ were less likely to report favourably in many areas of the hospital CERC. These findings suggest that there is a need to consider sociodemographic characteristics in relation to hospital CERC. Similar to planning for emergencies at national level or in cities, the presence of diverse populations in a setting or context needs to be taken into account [15]. In this instance, it will be important to engage and address the different population segments within the hospital to develop stronger partnerships with them in order to promote trust and actions in response to the COVID-19 pandemic. This is pertinent because doctors, and indeed all healthcare staff, are critical members in the hospital setting and in managing a public health crisis. When audience segmentation to which communication is directed at is insufficient and content is inadequate, it could have adverse consequences on compliance with recommended behaviours in CERC [14].

Communication challenges in translating action plans on the ground level and executing logistics planning and resource reallocation was also found to be a barrier in hospital CERC. Specifically, the role of middle management or local leadership to communicate and translate hospital updates to actionable plans was found to be crucial, least they become problematic to mitigating COVID-19 pandemic because of delayed or ineffective communication. A recent WHO commissioned study highlights that local leaders play vital roles in building trust and engaging an affected population [19]. Evidence also shows that providing CERC training workshops, tabletop exercises, simulations, and ensuring capacity building in this regard to enhance emergency communication response may help strengthen local leaders in their communication during public health crises [12, 28, 29]. Additionally, communicating plans from scenario modelling, which provide options for action [15] may clarify for its staff the hospital’s rationale for resource reallocation. This would be relevant in the implementation of patient screening points and scheduling of roster. Articulating mid- to longer term logistical plans such as in staff reallocation issues would be important.

A lack of communication on human resource matters to address staff’s welfare issues and concerns promptly was reported to have impacted staff morale and perceived support during this crisis. For example, in this study, staff reported that the level of empathy was lower than desired and being unable to take leave to rest during the initial stage of the crisis was putting their mental health at risk. Indeed, the statement ‘SGH has been able to understand my challenges and address my concerns during this outbreak’ had the lowest endorsement rate at 79.4% from all respondents among the questions. While the hospital provides psychological, counselling, and peer support services to its staff, communicating how staff could mitigate stress and directly addressing staff’s concerns of safety and well-being still remain crucial, which could help demonstrate empathy for its people [30, 31].

Even though emails as a communication tool may be useful, respondents reported that it does not necessarily provide timely release of information, particularly in a rapidly evolving situation such as COVID-19. This could affect adoption of recommended behaviours or adherence to instructions in CERC [11, 14]. The use of secure text messaging applications in hospital CERC may instead provide staff with more real-time updates. After all, it is crucial to always consider the circumstances in question and the target audience in communication [32]. In doing so, it would ensure an expeditious dissemination of information, and at the same time, address the needs of younger staff for more real-time updates. An anonymous feedback channel, albeit useful as proposed by some respondents, is generally not recommended as people should take ownership of their contributions, particularly in public health crises [33].

This study has demonstrated how CERC cuts across domains of hospital emergency response, such as command and control, communication, safety and security, triage, human resources, and logistics [21, 34, 35]. CERC transcends all these domains at various scales, which suggests the need for coordination and collaboration in CERC [29]. The interrelatedness of CERC and the domains of hospital emergency response highlight the need to consider communication at every domain of the hospital emergency response.

In essence, clear and accurate communication is necessary and indeed crucial for rapid, effective response to a critical event [35]. This study has shown that while this has been achieved thus far in SGH, there remains areas in which CERC could be enhanced within the hospital setting. Hospital CERC may mirror CERC when applied to the public in many aspects in terms of enablers and barriers, but population groups, sociodemographic factors and concerns may vary. For example, this study has identified and proposed the value of addressing different population segments based on sociodemographic characteristics in the hospital. It also suggests considering CERC in the context of hospital emergency response domains. Additionally, while SGH CERC may have met the general CERC principles of be first, be right, and be credible, areas such as expressing more empathy and showing respect for its staff, and addressing different population groups within the hospital to promote action are CERC principles that still need work. The findings above have informed how CERC can be better enhanced in the hospital in the subsequent phases, particularly as the COVID-19 pandemic continues to evolve. They may also have wider relevance to hospitals and countries elsewhere, which are combatting this crisis or other public health crises.

### Implications for future practice and policy

There are four key implications for future practice policy and policy. First, CERC is of importance and is critical to supporting efforts in managing and mitigating a public health crisis. Hospitals should consider providing CERC training workshops, tabletop exercise and simulations, particularly to strengthen local level leadership in communicating and translating actionable plans on the ground. In addition, a joint-hospital effort exercise at the national level will facilitate a focus on communication across healthcare institutions to mitigate new and emerging public health crises and the associated challenges. Second, there is a possible role for the national healthcare system to provide oversight and to ensure clear and effective CERC within and across healthcare institutions. This is because in public health crises, the country is dependent on its healthcare system’s ability to detect, mitigate and manage such situations effectively, and CERC is key to this process. Third, hospitals need to be cognizant of the sentiments on the ground, and a balancing act is required to ensure staff’s well-being, while operational needs and healthcare services are well looked into and addressed. An open, moderated communication channel would be essential and necessary. Lastly, identifying and creatively capitalizing on the best and most appropriate communication channels to address individual population segments, at various levels, and for a range of circumstances and will be helpful. Future studies on the subsequent phases of the CERC rhythm (maintenance and resolution) will be useful and may add to the learnings for future practice and policy.

### Study limitations

This study has several limitations. First, the cross-sectional nature of the study limits the ability to assess the temporal relationship of perceptions of crisis and emergency risk communication and sociodemographic characteristics over time. And while the study took place at the early days of the COVID-19 situation, when things were new and fast evolving and there could be a risk of a positive response bias, it is still relevant and valued. It would inform communication strategies in the management of COVID-19 in the subsequent phases (maintenance and resolution) of the CERC framework, particularly when little was known about the COVID-19 virus at the start, and when the hospital had to ensure dissemination of critical information and explaining risks to mitigate any safety risk concerns owing to the virus. Second, the self-reporting nature of the study also means that there could be a certain degree of social desirability in this study. Additionally, as non-probability sampling strategy was used in this survey, the sample obtained is subject to self-selection bias. This bias was exacerbated by the short duration of the survey due to the urgency of using the survey findings to inform hospital practice and policy on crisis and emergency risk communication, which may not leave adequate time to capture the attention of all hospital staff. As a result, our sample may not be representative of the hospital staff. In particular, doctors are under-represented in our study, and hence our study results cannot be generalised in terms of occupation.

## Conclusion

Right and timely communication at all levels can save lives in public health emergencies, and identifying the enablers and barriers can affect lives of staff and service-users [23]. While effective emergency communication is not a panacea for the challenges posed by any emergency public health threats, it is essential to help people maintain confidence and equip them with the necessary knowledge and attitude to cope with the situation.

This study has described the perception of crisis and emergency risk communication strategies used by an acute hospital in response to COVID-2019 in Singapore and the associated enablers and barriers. While regular updates were understandable and actionable, accurate, concise, and timely, and were clear and helpful, there was a need to consider segmenting population within the hospital and addressing them based on their sociodemographic characteristics such as job roles. In particular, it will be important to engage the doctors, younger staff and non-married staff to develop stronger partnerships with them to promote action in combating COVID-19. Use of secure text messaging in communication to provide real-time updates may be more useful than emails. Expressing more empathy and care for staff and supporting local leaders to communicate and translate updates to actionable plans would be pertinent. Better communication to execute resource reallocation plans and considering CERC in the context of hospital emergency response domains will be important.

As the COVID-19 situation evolves, there is a need to continuously evaluate the emergency risk communication. Health care systems and hospitals need to be sensitive to the factors that may enable or act as barrier to effective crisis and emergency risk communication with the goal of reducing and minimizing harm. This study has also provided a frame for hospitals to strategise CERC in combating COVID-19 and other public health crises.

## Supplementary Information

**Additional file 1.** CERC Survey. Crisis and Emergency Risk Communication Survey.

## Data Availability

Data can be requested from the corresponding author.

## Abbreviations

CDC: Centers for disease and control
CEO: Chief executive officer
CERC: Crisis and emergency risk communication
CMB: Chairman, medical board
COVID-19: Coronavirus disease 2019
SD: Standard deviation
SGH: Singapore general hospital
US: United States
WHO: World health Organisation
