# Supplementary material for: Perception survey of crisis and emergency risk communication in an acute hospital in the management of COVID-19 pandemic in Singapore
Source: BMC Public Health. 2020 Dec 17;20:1919. doi: 10.1186/s12889-020-10047-2 (PMC7745758; doi:10.1186/s12889-020-10047-2)
Supplement: Supplementary file 1 — Additional file 1. CERC Survey. Crisis and Emergency Risk Communication Survey. [file 12889_2020_10047_MOESM1_ESM.docx]

*Right and timely communication by the right people can save lives during a crisis or an emergency. This survey seeks to examine the communication strategies used in the management of the ongoing COVID-19 outbreak in SGH. Findings from this study will be used to inform communication planning in the management of current and future public health crises and emergencies. It will also make recommendations to enhance our response to the current COVID-19 outbreak management in terms of policy and practice.*

*Please check the answer that best describes your agreement with each statement. There is no right or wrong answer. Your response is anonymous and will be kept strictly confidential.*

|  | **Strongly**  **Disagree** | **Disagree** | **Neither Disagree Nor Agree** | **Agree** | **Strongly Agree** |
| --- | --- | --- | --- | --- | --- |
| The regular updates from SGH on the COVID-19 situation are understandable and actionable. | **** | **** | **** | **** | **** |
| SGH adequately prepares me for the challenges I am likely to face. | **** | **** | **** | **** | **** |
| The crisis communication plans are clear so far. | **** | **** | **** | **** | **** |
| SGH Senior Management possesses the necessary knowledge and expertise on the situation, and has been consistent in the delivery of their message. | **** | **** | **** | **** | **** |
| The information released by SGH senior management has been accurate, concise and timely, and are repeated enough to keep staff safe. | **** | **** | **** | **** | **** |
| My direct superior has consistently provided me with accurate, concise and timely information for me to navigate in this disease outbreak. | **** | **** | **** | **** | **** |
| I am sufficiently engaged in the preparedness planning. | **** | **** | **** | **** | **** |
| SGH has been able to understand my challenges and address my concerns during this outbreak. | **** | **** | **** | **** | **** |
| SGH has been able to provide explanations of the risks associated with the COVID-19 situation in a simple, concise and direct manner. | **** | **** | **** | **** | **** |
| SGH has been clear in explaining the necessary actions I need to take to stay safe. | **** | **** | **** | **** | **** |
| I am clear about what SGH is doing in response to the COVID-19 situation. | **** | **** | **** | **** | **** |
| The constant updates from SGH Senior Management increase my trust in the credibility of SGH. | **** | **** | **** | **** | **** |
| Platforms, such as emails and social media (Facebook Workplace group – ‘Pneumonia (China) – Chat with CEO & CMB’) provide useful avenues for sharing of information and feedback. | **** | **** | **** | **** | **** |

**Background Information**

1. Are you a/an:

** **Doctor ****Nurse ****Allied Health Professional ****Administrative staff member

****Ancillary support staff member – SGH employees

****Ancillary support staff member – External staff contracted to work in SGH

1. Do you have staff reporting to you?

****No ****Yes

1. Where do you obtain your information and updates on the management of COVID-19 in SGH? (Please select all that apply)

****SingHealth Staff Advisory

****SGH Senior Management updates, guidelines and instructions

****Social media (e.g., SGH Facebook Workplace Group)** **

****Supervisor

****SGH Middle Management (e.g., Head of Department, Division Director)** **

****Department emails

****Colleagues and peers (e.g., chat groups, exchanges)

****Others (please specify: ________________________)

1. Does your work require you to have direct contact with patients?

****No ****Yes

1. What is your primary work location since the start of the COVID-19 outbreak?

****Emergency Department ****Inpatient – Isolation ward(s)

****Inpatient – Other clinical area(s)**      **Outpatient ****Non-clinical area(s) (no patient contact)

1. Are you directly involved in treating COVID-19 or suspected COVID-19 cases?

****No ****Yes

1. Gender

****Female ****Male

1. Age in years _____
2. Years of working experience in SGH _____
3. Nationality

****Singaporean or PR ****Malaysian ****Filipino ****Indian ****Chinese

****Myanmar ****Other nationalities (please specify: ____________________)

1. Marital status

****Single ****Married ****Separated or Divorced ****Widowed

1. What do you think of this survey and how do you think we can improve it?
